# Correction: Speaking out of turn: How video conferencing reduces vocal synchrony and collective intelligence

**DOI:** 10.1371/journal.pone.0287800

**Published:** 2023-06-23

**Authors:** Maria Tomprou, Young Ji Kim, Prerna Chikersal, Anita Williams Woolley, Laura A. Dabbish

There are errors in the Funding statement. The correct Funding statement is as follows: This material is based upon work supported by the National Science Foundation under grant numbers CNS-1205539 (url: https://www.nsf.gov/awardsearch/showAward?AWD_ID=1205539&HistoricalAwards=false) and OAC-1322278 (url: https://nsf.gov/awardsearch/showAward?AWD_ID=1322278) (Author who received the awards: L.D.). This study was also supported by the Defense Advanced Research Projects Agency and the Army Research Office Grant Number W911NF-20-1-0006 (Author who received the award: A.W.). The views and conclusions contained in this document are those of the authors and should not be interpreted as representing the official policies, either expressed or implied, of the Defense Advanced Research Projects Agency, the Army Research Office, or the U.S. Government. The U.S. Government is authorized to reproduce and distribute reprints for Government purposes notwithstanding any copyright notation herein.
