# Supplementary material for: Speaking out of turn: How video conferencing reduces vocal synchrony and collective intelligence
Source: PLoS One. 2021 Mar 18;16(3):e0247655. doi: 10.1371/journal.pone.0247655 (PMC7971580; doi:10.1371/journal.pone.0247655)
Supplement: S1 Appendix — (PDF) [file pone.0247655.s001.pdf]

S1 Appendix

t-test results Comparing Cases with Valid and Missing Data

Demographic		Mean	(SD)	t
Social perceptiveness	Valid	26.25	(2.87)	1.74
	Missing	27.11	(2.81)	
Female number	Valid	.98	(.83)	.51
	Missing	1.01	(.73)	
Age distance	Valid	5.64	(7.59)	1.56
	Missing	8.82	(12.62)	
Education distance	Valid	1.22	(1.10)	.37
	Missing	1.30	(1.25)	
Ethnic similarity	Valid	.36	(.48)	-.27
	Missing	.34	(.47)	

Note. Valid = 99 dyads; Missing = 47 dyads.
