# Supplementary material for: Speaking out of turn: How video conferencing reduces vocal synchrony and collective intelligence
Source: PLoS One. 2021 Mar 18;16(3):e0247655. doi: 10.1371/journal.pone.0247655 (PMC7971580; doi:10.1371/journal.pone.0247655)
Supplement: Attachment — Submitted filename: Response to Reviewers_ PONE-D-20-24495.docx [file pone.0247655.s002.docx]

PONE-D-20-24495

Visual Cues Disrupt Prosodic Synchrony and Collective Intelligence in Distributed Collaboration

PLOS ONE

Dear Dr. Tomprou,

Thank you for submitting your manuscript to PLOS ONE. After careful consideration, we feel that it has merit but does not fully meet PLOS ONE’s publication criteria as it currently stands. Therefore, we invite you to submit a revised version of the manuscript that addresses the points raised during the review process.

My apologies for the delay getting your manuscript reviewed. I have now received reviews from three referees with various expertise on the topic of your study. All three reviewers are positive about the work, with two recommending acceptance. The second reviewer, however, notes some questions for clarification and offers some constructive suggestions. I agree with the reviewer that these points need to be addressed in order for the manuscript to be suitable for publication. Please pay particular attention to the questions raised about details relating to the methodology of the study.

Please submit your revised manuscript by Jan 08 2021 11:59PM. If you will need more time than this to complete your revisions, please reply to this message or contact the journal office at plosone@plos.org. When you're ready to submit your revision, log on to https://www.editorialmanager.com/pone/ and select the 'Submissions Needing Revision' folder to locate your manuscript file.

Please include the following items when submitting your revised manuscript:

- A rebuttal letter that responds to each point raised by the academic editor and reviewer(s). You should upload this letter as a separate file labeled 'Response to Reviewers'.
- A marked-up copy of your manuscript that highlights changes made to the original version. You should upload this as a separate file labeled 'Revised Manuscript with Track Changes'.
- An unmarked version of your revised paper without tracked changes. You should upload this as a separate file labeled 'Manuscript'.

If you would like to make changes to your financial disclosure, please include your updated statement in your cover letter. Guidelines for resubmitting your figure files are available below the reviewer comments at the end of this letter.

If applicable, we recommend that you deposit your laboratory protocols in protocols.io to enhance the reproducibility of your results. Protocols.io assigns your protocol its own identifier (DOI) so that it can be cited independently in the future. For instructions see: <http://journals.plos.org/plosone/s/submission-guidelines#loc-laboratory-protocols>

We look forward to receiving your revised manuscript.

Kind regards,

Marcus Perlman, Ph.D

Academic Editor

PLOS ONE

**Authors’ Response: Thank you for the opportunity to revise and resubmit our paper. We hope we have now carefully and successfully addressed all the comments**

Journal Requirements:

When submitting your revision, we need you to address these additional requirements.

1. Please ensure that your manuscript meets PLOS ONE's style requirements, including those for file naming. The PLOS ONE style templates can be found at

https://journals.plos.org/plosone/s/file?id=wjVg/PLOSOne_formatting_sample_main_body.pdf and

<https://journals.plos.org/plosone/s/file?id=ba62/PLOSOne_formatting_sample_title_authors_affiliations.pdf>

**Response: After careful editing, we believe we have met all PLOS ONE’s style requirements.**

2. Please ensure that you refer to Figure 4 in your text as, if accepted, production will need this reference to link the reader to the figure.

**Response: We apologize for omitting the reference in the actual text. We now have properly referenced all of our Figures.**

3.We note that Figure [1] includes an image of a patient / participant in the study.

As per the PLOS ONE policy (http://journals.plos.org/plosone/s/submission-guidelines#loc-human-subjects-research) on papers that include identifying, or potentially identifying, information, the individual(s) or parent(s)/guardian(s) must be informed of the terms of the PLOS open-access (CC-BY) license and provide specific permission for publication of these details under the terms of this license. Please download the Consent Form for Publication in a PLOS Journal (http://journals.plos.org/plosone/s/file?id=8ce6/plos-consent-form-english.pdf). The signed consent form should not be submitted with the manuscript, but should be securely filed in the individual's case notes. Please amend the methods section and ethics statement of the manuscript to explicitly state that the patient/participant has provided consent for publication: “The individual in this manuscript has given written informed consent (as outlined in PLOS consent form) to publish these case details”.

If you are unable to obtain consent from the subject of the photograph, you will need to remove the figure and any other textual identifying information or case descriptions for this individual.

**Response: We have obtained participant’s signed consent and we have added the related statement in the Method section (see lines 118-119).**

4. We noted in your submission details that a portion of your manuscript may have been presented or published elsewhere.

[A pilot of this study was presented at the Computer Supported Cooperative Work and Social Computing Conference in 2017 (please the Related Manuscript). Since that time we have collected additional data, including the addition of Condition 2 in which participants collaborated only via audio. (please see the table of the cover letter for overlapping variables).]

Please clarify whether this [conference proceeding or publication] was peer-reviewed and formally published. If this work was previously peer-reviewed and published, in the cover letter please provide the reason that this work does not constitute dual publication and should be included in the current manuscript.

**Response: Please see our detailed response in the cover letter.**

Reviewer #1: This is a thoughtful, elegant manuscript that meets (and exceeds) the bar for publication--and also offers really interesting, counterintuitive, and practical implications. It is technically sound, and the authors are clearly experts in this field. Excellent submission.

**Response: Thank you for your kind words.**

Reviewer #2: #Review of manuscript PONE-D-20-24495 for PLOS ONE

The authors of this work report the results of an experimental study in which they investigate the verbal and nonverbal behaviour and task performance of dyads who carry out several tasks while in different locations (“distributed collaboration”) and connected via audio-only or using concurrent auditory and visual modalities (through live video). They observe that the degree of prosodic synchrony within dyads in the audio-only condition was higher compared to the degree of prosodic synchrony in the audio+video group. No overall differences in collective intelligence measures and facial expression synchrony were observed between the two groups/conditions, but higher prosodic synchrony correlated with higher collective intelligence. The authors argue that the presence of video may thus not only have benefits (as suggested by previous work and anecdotal evidence) but also drawbacks for communication and collaboration, and highlight the societal relevance of these findings in the current digital era. This research is timely, the manuscript is well-written, and I agree that the findings have clear societal relevance. Several questions for clarification keep me from recommending acceptance of the manuscript in its current form.

**Response: Thank you for your kind comments and also for your suggestions to improve our manuscript toward publication.**

Minor comments and suggestions

1. The word ‘disrupts’ in the title is quite strong in the absence of a causal effect of the presence of video and in light of the finding that “there was not a significant difference in the observed level of collective intelligence” across the two conditions/groups (line 224).

**Response**: **This is an excellent point, and we thank you for raising it. In reconsidering the title based on your feedback we have decided to alter it to more accurately reflect the chain of relationships we observe in the data. This results in a slightly longer but more accurately descriptive title which we hope you will agree is more appropriate.**

**The new title is: “Speaking out of turn: How video conferencing reduces vocal synchrony and collective intelligence”**

2. The authors theoretically contrast their set-up with earlier studies that investigated similar issues in ‘face-to-face’ set-ups (see e.g. the abstract, and line 46-48). However one could argue that also in the authors’ video condition, participants are sitting face-to-face – they are just in different locations (see also our description in the laboratory published [protocol](http://dx.doi.org/10.17504/protocols.io.bqfamtie)). I would therefore suggest being more careful when using the term ‘face-to-face’ or avoiding it at all.

**Response:** **Thank you for raising this point, which highlighted for us the need to clarify our procedure. In our study, participants were taken to physically separated rooms from the very beginning of the experiment (see also the subsection Participant Recruitment and Data Collection, lines 121- 134), and could only see and/or hear each other via the small screen-based video-conference window and audio available via their computer. In the literature, “face-to-face” interactions refer to physically collocated interactions not mediated in any way by technology (e.g., Crowley and Mitchell, 1994 ; Nardi & Whitaker, 2002) which allow collaborators to access much more nonverbal cues than simply seeing a fairly low-resolution video of their collaboration partner’s face. So we believe our study does appropriately operationalize a remote collaboration scenario distinct from face-to-face setups investigated in prior research.**

**Crowley, D. J., & Mitchell, D. (Eds.). (1994). *Communication theory today*. Stanford University Press.**

**Nardi, B. A., & Whittaker, S. (2002). The place of face-to-face communication in distributed work. *Distributed work*, *83*, 112.**

3. The work by Levinson on turn-taking is relevant to the discussion of turn-taking in the current manuscript and I would suggest referring to it as it has been highly influential. See for instance:

Levinson, S. C. (2016). Turn-taking in human communication–origins and implications for language processing. Trends in Cognitive Sciences, 20(1), 6-14.

**Response: Thank you for suggesting this important reference. We have now incorporated Levinson’s work into our discussion of turn-taking. Please see line 49.**

4. The authors’ focus on synchronization of facial expressions (line 79) comes a bit as a surprise to the reader at this point in the text. I would suggest adding a paragraph prior to the current line 79 in which earlier work on facial expression synchrony is discussed more explicitly.

**Response:** **Thank you for your comment. In thinking more about this, we now introduce synchrony in facial expressions (and vocal cues) in lines 31-33.**

5. The authors explain that they used six tasks to measure collective intelligence. More information is needed here in the main text. Which six tasks were used exactly and what was the procedure used in each task? What number (mean unweighted score) corresponds to a high score on those tasks and what corresponds to a relatively low score?

**Response**: **We used six tasks (typing, matrix solving, sudoku, unscramble words, memory, brainstorming) that represent executing, remembering, generating, and choosing, respectively. Participants were logged onto the Platform for Online Group Studies (POGS) with the username Participant A or Participant B and worked synchronously together on each of the tasks. The POGS system was used to automatically administer the tasks (e.g., presenting instructions and task interface, regulating time). We provide detailed information for the procedure in our procedure section as well as in our published** [**protocol**](http://dx.doi.org/10.17504/protocols.io.bqfamtie)**. In addition, we provide a table describing the nature of tasks, scoring rules, and descriptive statistics of task scores (see file in our protocol and below for your convenience).**

| **Task** | **Process** | **Description** | **Scoring** | **Score Statistics** |
| --- | --- | --- | --- | --- |
| Typing Text | Executing | Collectively create a copy of a text as accurately as possible | Get a point for a word correctly typed while getting penalty for holes, typos, misplaced words | M: 227.93  SD: 108.97  Min: 17.97  Max: 592.16 |
| Sudoku | Executing | Collectively solve a sudoku puzzle where participants have to fill a 9 x 9 grid with digits so that each column, row, and 3x3 sub-grid contains 1 to 9. | Get a point for a correctly placed number | M: 14.65  SD: 8.20  Min: 1  Max: 42 |
| Matrix Solving | Choosing | Collectively solve matrix puzzle problems | Get a point for a correct answer | M: 8.29  SD: 2.25  Min: 2  Max: 13 |
| Unscramble Words | Choosing | Collectively figure out what word is represented in a set of letters which have been randomly shuffled around | Get a point for a correct answer | M: 5.31  SD: 1.85  Min: 1  Max: 12.5 |
| Brainstorming Brick | Generating | Collectively come up with as many unique uses for brick as possible | Get a point for each unique idea | M: 8.63  SD: 3.32  Min: 0  Max: 19 |
| Memory Picture | Remembering | Collectively remember enough about a busy picture and answer specific questions about the picture | Get a point for a correct answer | M: 2.80  SD: 1.19  Min: 0  Max: 6 |

6. In the absence of more explicit information about the tasks that were used, I found it hard to understand why speaking turn inequality (see section 2.2.5) would be a theoretically relevant measure/proxy of a dyad’s spoken communication quality/performance. I would clarify more extensively early on in the manuscript that it has previously been related to collective intelligence, as is now explained in lines 251-252.

**Response: We describe in greater detail now previous findings associating speaking equality and collective intelligence. Please see lines 44-51.**

7. Very minor stylistic comments: the words co-located and collocated are used interchangeably throughout the manuscript. The final sentence of the abstract seems ungrammatical.

**Response: We have now consistently used the word collocated. The verb “call” refers to the findings and not to the non-verbal synchrony so we believe the sentence is correct in the last sentence of our abstract.**

Reviewer #3: The authors investigate the effect of utilizing video versus non-video (audio only) communication on collective intelligence in dyads. Their investigation is nuanced, analyzing the dynamics of mediating and moderating factors in this process.

**Response: Thank you.**

The design of the experiment is clearly explained, and is sound. The same is true of the analytical strategy.

**Response: Thank you.**

The results of the study are counterintuitive, but are well-explained by the authors. The idea that the presence of video may actually hinder communication is a fascinating one, and the implications are important. As the authors mention in their discussion of future directions, it will be interesting to see whether these results generalize to larger groups.

**Response: Thank you and we agree about future research in larger working groups.**

In the discussion section, I would like to see a little more discussion/elaboration on the finding displayed in Figure 3. If video (versus audio only) may increase the importance of social perceptiveness (due to its effect on facial expression synchrony), this is an important dynamic for people and organizations to take into consideration.

**Response: Dear Reviewer, thank you for the kind feedback. Related to your specific comment here we have now elaborated on our findings related to Figure 3 and connected with some relevant research (please see lines 279-285). Thank you!**

I enjoyed reading this work - best of luck to all of the authors in the future!

**Response: Thank you!**
